# Bilateral Involvement in Developmental Dislocation of the Hip: Analysis of 561 Patients Operated on Using the Limited Posteromedial Approach

**DOI:** 10.3390/children11010037

**Published:** 2023-12-28

**Authors:** Batuhan Gencer, Özgür Doğan, Ali Biçimoğlu

**Affiliations:** 1 Department of Orthopaedics and Traumatology, Sancaktepe Şehit Prof. Dr. İlhan Varank Training and Research Hospital, 34785 Istanbul, Turkey; 2 Department of Orthopaedics and Traumatology, Ankara Bilkent City Hospital, 06800 Ankara, Turkey; ozgur.dogan5@saglik.gov.tr (Ö.D.); ali.bicimoglu@saglik.gov.tr (A.B.)

**Keywords:** developmental dysplasia of the hip, bilateral involvement, limited posteromedial approach, risk factor, odds ratio, redislocation, avascular necrosis

## Abstract

Our objective was to scrutinize the risk factors related to bilateral involvement in the developmental dysplasia of the hip (DDH) and to inspect the impact of bilaterality on the enduring results of the DDH. All patients, aged between 6 and 18 months, who underwent surgery using the limited posteromedial approach (734 hips from 561 patients), were included in this study. The number of births, birth type, history of consanguineous marriage, family history, and swaddling were analyzed. Physical examination and complaints of the patients were evaluated, and direct radiographs were examined in terms of the redislocation, avascular necrosis, and residual acetabular dysplasia. Among the 561 patients, bilateral DDH was observed in 173 patients (30.8%). The use of swaddling was found to be statistically significant between groups (*p* = 0.012). The use of swaddling for more than one month was associated with a higher odds ratio for bilaterality (*p* = 0.001, OR = 1.56, 95% CI: 1.2–2.0). Furthermore, bilaterality was associated with a higher risk for redislocation in DDH (*p* = 0.001, OR = 4.25, 95% CI: 1.6–11.2). The study concludes that swaddling for over a month is strongly linked with the bilateral involvement in DDH. It is important to note that bilaterality plays a crucial role in the development of redislocation after open reduction in DDH.

## 1. Introduction

Developmental dysplasia of the hip (DDH) is a condition characterized by the disruption of the connection between the femoral head and acetabulum during hip joint development. It is a significant cause of disability worldwide. The condition may manifest during the postnatal development of the hip joint, based on the common contributing factors. Early diagnosis and appropriate treatment in the first few months of life can greatly reduce the morbidity associated with this hip problem. Late diagnosis, delayed treatment, and inappropriate medical care heighten the probability of developing degenerative joint disease in the hip. The management of the developmental dysplasia of the hip is determined based on the age at which it was diagnosed. In infants under six months of age, treatment through the use of a Pavlik harness is adequate. In children over six months of age, closed reduction under general anesthesia is recommended, and open reduction is required to remove any interfering soft tissues and to ensure the femoral head is placed concentrically in the acetabulum in cases where unforced closed reduction is not possible. In children older than two years of age, pelvic and femoral osteotomies should be added to the treatment. Regardless of the treatment method applied, all patients should be followed up until skeletal development is completed to determine complications and to evaluate the necessity of secondary operations [1,2,3,4,5].

There are many studies in the literature investigating the factors affecting complications after developmental hip dysplasia. Redislocation, residual acetabular dysplasia (RAD), and avascular necrosis of the femoral head (AVN) are among the most prevalent complications following developmental dysplasia of the hip (DDH). By identifying the factors that contribute to these complications and adopting preventative measures, one can mitigate the morbidity and disability associated with DDH [6,7,8,9]. In a recent study performed, it was determined that bilateral involvement is an important risk factor for redislocation [7]. Over the years, researchers have found that bilateral involvement is associated with redislocation, residual acetabular dysplasia, and avascular necrosis. However, these studies have certain limitations, such as a short follow-up period, a limited number of patients, and an evaluation of only patients who have reached walking age or neglected and late diagnosed cases [10,11,12,13]. From this point of view, we hypothesized that bilateral involvement affected clinical outcomes, the necessity of secondary operation, and complication rates over a long-term follow-up period.

Bilaterality is also important in the diagnostic process of DDH. When patients with suspected hip dislocation are evaluated by less experienced surgeons, comparative radiographs are commonly used. On the other hand, bilateral cases may complicate this diagnostic process. By identifying the risk factors that cause bilaterality, high-risk cases can be identified and evaluated more clearly. Unfortunately, to the best of our knowledge, there are a limited number of studies investigating the risk factors that affect bilateral involvement in DDH, and there is no consensus in the literature regarding the risk factors that affect bilateral involvement. Okano et al. in 2008 and Simonsen et al. in 2018 established a relationship between positive family history and bilateral involvement and argued that genetic predisposition increases bilaterality [11,14]. However, Loder and Schafer reported in 2015 that they did not find any relationship between family history and bilaterality [15]. The literature suggests that bilateral involvement may also be associated with race, ethnicity, and geography [14,16]. Additionally, the relationship between breech presentation and bilaterality has been investigated in the literature, but no definitive conclusion has been reached regarding whether the relationship is due to genetic predisposition or mechanical factors [14,16,17].

The primary aim of the investigation was to scrutinize and evaluate the risk factors related to bilateral involvement in DDH. The ancillary goal was to inspect the impact of bilaterality on the enduring results of DDH patients who received treatment using the limited posteromedial approach.

## 2. Materials and Methods

**Figure 1:**
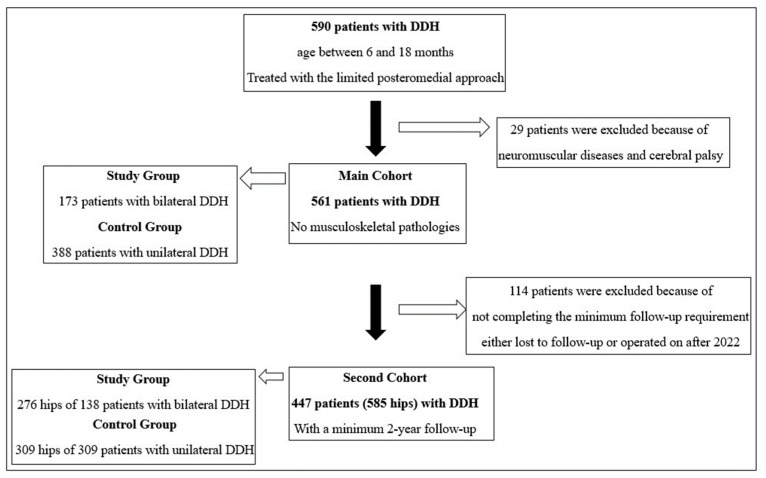
Schematic description of the patients of the study and control groups in the main and second cohorts.

In our clinic, patients with DDH between 6 and 18 months have been treated by the same senior surgeon (AB) with the limited posteromedial approach since 1993. These patients were followed up regularly and prospectively, and all patients with any disease that may affect the musculoskeletal system (neuromuscular disease, cerebral palsy, etc.) were excluded from the patient series. Following ethics committee approval, all 561 patients with DDH, aged between 6 and 18 months and treated with open reduction via the limited posteromedial approach, were evaluated, regardless of the minimum follow-up requirement. To evaluate the long-term outcomes of bilaterality, a second cohort consisting of patients with DDH, aged between 6 and 18 months, treated with open reduction via the limited posteromedial approach, and who had at least two years of regular follow up (585 hips from 447 patients), was formed and analyzed (Figure 1).

All patients were operated on with the limited posteromedial approach, as described in the literature [18,19,20,21]. The limited posteromedial approach is a surgical approach that was defined in 1993 and has been applied since then, and its short-, medium-, and long-term results have been reported previously [19,20,21]. In this modified approach, the hip capsule is not routinely opened and a “limited” approach is used instead. In this modified limited approach, hip stability is evaluated via perioperative hip arthrography after the removal of extracapsular obstacles, such as adductor tenotomy and the release of the iliopsoas muscle, and the operation is terminated without opening the hip capsule in cases with adequate stability. If, according to the results of the perioperative arthrography, the pooling of the contrast material was detected medially and/or the hip was lateralized, the hip capsule was opened and the ligamentum teres were excised. No preliminary traction was used in any patients. In cases of bilateral DDH, surgery was performed on both sides during the same session and no staged surgery was carried out. In the postoperative period, regardless of whether the hip capsule was opened or not, all patients were followed in the human position for the first three months with a hip spica cast and then with a rigid abduction orthosis (24 h a day) for another three months. All patients’ hips were released at six months postoperatively, and they were then called for annual controls until the age of 10 and every two years until bone development was completed. Physical examination, active gait patterns, and complaints of the patients were evaluated at each outpatient clinic control, and direct radiographs were examined in terms of redislocation, avascular necrosis, and residual acetabular dysplasia. No advanced imaging techniques other than direct radiography were used.

As mentioned before, a main cohort, regardless of the minimum follow-up time requirement, was established to investigate the effects of known DDH-related risk factors on bilaterality. This cohort was divided into two subgroups: unilateral and bilateral cases. Aside from demographic information such as age and sex, the relationship between bilaterality and the number of births of the mother, history of the consanguineous marriage of the parents, family history for DDH, patient’s labor characteristics (normal delivery vs. caesarean section), and swaddling were examined. Patients with breech presentation and dystocia were included in the cesarean delivery group. Considering the sociocultural habits of our country, consanguineous marriage was categorized as “First Degree” if the parents were cousins of each other, and “Second Degree” if one of the spouses was the grandchild of the other’s grandmother or grandfather’s sibling. Family history in terms of DDH was categorized as “First-Degree” family history if it was present in the patient’s mother, father, or sibling, and as “Other” if it was present in any other relative.

The second cohort, formed with the requirement of a minimum two-year follow up, was analyzed to examine the long-term consequences of bilaterality. In addition to demographic data such as sex and age at the time of the open reduction, the follow-up period, the requirement of opening the capsule during surgery, the presence of RAD or AVN at the last follow up, the rate of all secondary operations (soft tissue and bone), regardless of the cause, the number of cases with redislocation (regardless of the treatment method), and the number of cases who required osteotomies, were evaluated. For risk analysis and odds ratio calculation, the rate of secondary surgery, redislocation rate, and osteotomy requirement were evaluated separately and two-by-two tables were created. For patients with redislocation, the routine treatment protocol in our clinic is to change the hip-spica cast under general anesthesia. If adequate stability cannot be achieved perioperatively or in recurrent redislocation cases, open reduction is repeated, and in a few cases where stability cannot be achieved and redislocation persists, osteotomy procedures are performed, as suggested and discussed in the literature [7].

IBM^®^ SPSS^®^ Statistics v.26.0 was utilized to conduct the statistical analyses. The conformity of the variables to the normal distribution was checked through the use of analytical (Kolmogorov–Smirnov test) and visual (histogram graphs) techniques. Given that all the continuous variables were skewed distributed, the median, interquartile range, and minimum–maximum range values were used as descriptive statistics, and the Mann–Whitney U test was used to compare variables. For categorical variables, frequencies and percentiles were preferred as descriptive statistics, and the chi-square test and Fischer’s exact tests were performed to compare variables. Fischer’s exact test was preferred in cases where the chi-square assumption was not met. A *p*-value lower than 0.05 was considered statistically significant. For risk analyses of the variables that were found to be significant, two-by-two tables were formed, and the Odds Ratio (OR) and 95% confidence interval (CI) were calculated.

## 3. Results

Among the main cohort of 734 hips of 561 patients, bilateral developmental hip dysplasia was observed in 173 patients, with the bilateral involvement rate of 30.8%. Among the second cohort of 585 hips from 447 patients, bilateral developmental hip dysplasia was observed in 138 patients, with the bilateral involvement rate of 30.9%.

Despite finding no significant relationship between patient’s sex, the number of births of the patient’s mother, birth type, family history of hip dysplasia, consanguineous marriage between parents, and bilateral involvement (*p* < 0.05), the use of swaddling was found to be statistically significant between groups in the main cohort (*p* = 0.012). Further risk analysis revealed that the use of swaddling for more than one month was associated with a higher odds ratio for bilateral involvement (OR = 1.56, 95% CI: 1.2–2.0). The risk analysis of the main cohort for bilateral involvement can be seen in detail in Table 1.

With a minimum follow-up period of two years and a mean follow-up period of 9 years (range, 2–25 years), there was a statistically significant difference in the prevalence of redislocation between the bilateral and unilateral groups in the second cohort (*p* = 0.001). Further risk analysis revealed that bilateral involvement was associated with a higher odds ratio for redislocation in DDH (OR = 4.25, 95% CI: 1.6–11.2). On the other hand, no significant relationship was observed between bilateral involvement and residual acetabular dysplasia, avascular necrosis, necessity for secondary surgery, or necessity for osteotomy. In addition, in four patients (16.7%) who underwent redislocation, osteotomy was performed in the long-term follow up. Refer to Table 2 for a comparison of the clinical outcomes of the hips between the groups in the second cohort.

## 4. Discussion

Developmental hip dysplasia is a congenital deformity of the hip joint that can be detected early in life. Early diagnosis and proper treatment can prevent catastrophic complications. To the knowledge of the researchers, there are a limited number of studies in the literature investigating the risk factors affecting the development of bilateral involvement in DDH [11,12,13,14,15,16,17]. Additionally, few studies have examined the impact of bilaterality on long-term outcomes [10,11,12,13]. This study differentiates itself in these regards. The primary contribution of our study lies in being the first to focus on researching the factors influencing bilateral involvement in DDH, with a large series of patients. Furthermore, the study highlights the effects of bilateral involvement on clinical outcomes and complications in the long-term follow up of 9 years (range, 2–25 years). A key feature of the study is the identification of a link between swaddling and bilaterality (*p* = 0.012). Swaddling for more than a month is an important risk factor for bilateral involvement in DDH (*p* = 0.001, OR = 1.56, 95% CI: 1.2–2.0). In addition, although bilaterality was not found to be significantly associated with the need for secondary surgery, RAD, or AVN in the long-term follow up (*p* < 0.05 for each), it was found to have a significant relationship with redislocation (*p* = 0.001, OR = 4.25, 95% CI: 1.6–11.2).

Bilateral involvement in DDH was reported in 30.8–30.9% in this study. Different rates of bilateral involvement in DDH have been reported in the academic literature. A study conducted in Brazil in 2020 reported a bilaterality rate of 46.2% in DDH cases [22]. A Japanese study from 2017 reported the rate of bilateral involvement as 4% [23]. In 2016, a study in Jordan reported a bilateral involvement rate of 60.5% in Jordanian men [24]. Studies conducted in our country report bilateral involvement rates ranging from 22 to 29% in DDH cases [7,8,21]. Regional differences in the incidence of bilateral involvement in patients with developmental dysplasia of the hip imply that racial and genetic factors may contribute to this parameter. It is, therefore, imperative to conduct nationwide multicenter studies for a comprehensive investigation into this area. On the other hand, sociocultural factors, such as tight swaddling, could also influence the incidence of bilateral involvement. Swaddling is a traditional practice of wrapping a baby’s body gently in a light breathable blanket to promote calmness and sleepiness. It is one of the world’s most universal childcare practices and is becoming increasingly popular in several countries. In general, swaddled infants wake less and sleep longer, and swaddling can relieve infant pain and help regulate temperature. On the other hand, swaddling has been reported to pose several risks. In addition to the fact that swaddling in the prone position increases the risk of sudden infant death syndrome, and tight swaddling increases the risk of respiratory infections, it has been well known since the 1960s that swaddling with the legs in extension and adduction increases the risk of developmental hip dysplasia [25,26,27]. Salter first reported the association between swaddling and DDH and stated the role of geographical and cultural differences in different swaddling techniques and their effects on the development of DDH [27]. Considering that the study aimed to investigate the effect of known DDH-related risk factors on bilateral involvement, we have examined the relationship between swaddling and bilaterality. In fact, this research demonstrates a significant correlation between swaddling and bilaterality (*p* = 0.012). The risk of bilaterality increases in cases where swaddling is used for over a month (*p* = 0.001, OR = 1.56, 95% CI: 1.2–2.0).

Developmental dysplasia of the hip is a multifactorial, and primarily a developmental, deformity, with a genetic and hormonal basis, and many risk factors have been identified [2]. In this study, the relationship between these risk factors and bilateral involvement was investigated, and as mentioned before, we found a significant relationship between swaddling and bilateralism (*p* = 0.012). Further risk analysis revealed that the risk of bilaterality increases in infants who were swaddled for over a month (*p* = 0.001, OR = 1.56, 95% CI: 1.2–2.0). It is crucial to acknowledge the clinical implications of this finding, as it highlights the need for correct patient information. Namely, it is recommended that parents be made aware of the potential risks associated with swaddling, specifically that prolonged swaddling not only leads to the development of DDH but also increases the risk of bilateral involvement. Another important point to emphasize is that, as mentioned before, comparative radiographs are important in the diagnostic process of DDH, especially for less experienced surgeons. The fact that swaddling is known to increase bilateral involvement should be a warning for the clinician to be more careful when examining comparative radiographs of an infant who was swaddled.

Although there are several studies in the literature investigating long-term complications after DDH, studies focusing on the complication rates of cases with bilateral involvement are limited [10,11,12,13]. On the other hand, bilateral hip dislocation may be associated with poorer clinical outcomes due to more complex diagnostic process, difficulties in managing the treatment process, difficulties in positioning during casting after open reduction, and decreased family compliance. Based on these points, this study sought to examine the impact of bilateral involvement on long-term outcomes, with an average follow-up duration of 9 years (range: 2–25 years), and to identify any poor prognostic effects. We hypothesized that the incidence of complications and the need for secondary surgery would be higher in bilateral cases. There were several studies in the literature that supported our hypothesis. A 2022 study explored the development of redislocation in patients with DDH who underwent surgery via the limited posteromedial approach and reported that bilateral involvement emerged as a critical risk factor for redislocation [7]. Similarly, in 2011, an analysis indicated that bilateral involvement is a risk factor for redislocation after open reduction [28]. In their study in 2013, in which they examined 212 DDH patients of walking age, Wang et al. reported that bilateral involvement was a risk factor for the development of AVN [13]. In their retrospective evaluation in 2008, Okano et al. found a relationship between bilateral involved patients and acetabular dysplasia [11]. However, the findings of this study, in which the long-term results of a large series of patients were reported, contradict the literature. It was discovered that there is a significant correlation solely between bilateral involvement and the emergence of redislocation (*p* = 0.001, OR = 4.25, 95% CI: 1.6–11.2), as previously demonstrated. On the other hand, there was no significant relationship identified between the bilaterality and the need for capsule opening during surgery, the RAD or AVN rates at the last follow up, the necessity for secondary surgery, or the need for osteotomy (*p* > 0.05 for each). Upon considering the reasons for this finding, the first point that draws attention is that several studies in the literature reporting the poor prognostic effects of bilateral involvement were conducted in patient populations of walking age [10,11,13]. In this study, on the other hand, the average age of the patients at the time of surgery was 11 months (range: 6–18 months), and there was no significant difference in age between the groups (*p* = 0.469). This eliminates the difference of bilateral cases being operated on later, due to unilateral cases being recognized earlier. In addition, contrary to several studies in the literature [12], this study did not focus on neglected bilateral cases and analyzed patients who were diagnosed and treated in the early period. Another important point to emphasize is that bilateral involvement is not a genetic or hormonal risk factor but rather a mechanical one. The fact that bilateral involvement was linked to swaddling but not to factors like family history, sex, or consanguineous marriage also suggests that this parameter is a mechanical risk factor. In the literature, the association between bilateral involvement and redislocation has been explained via several mechanistic predictions, such as the challenges that arise during casting and positioning [7,28,29]. Biomechanical studies or multicenter research on this topic may provide more in-depth results.

When considering the potential applications of this research and its implications for clinical practice, it is immediately apparent that informing families about the risks associated with swaddling infants is of utmost importance. It is crucial that families receive adequate information regarding the fact that swaddling is linked not only to the development of DDH but also to bilateral involvement. Furthermore, it is crucial for the clinician to take into account the increased possibility of bilateral involvement in patients with a swaddling history during the diagnostic procedure and analyze comparative radiographs from this viewpoint. Furthermore, orthopedic surgeons must exercise caution regarding the 4.25-fold increased risk of redislocation during treatment of bilaterally involved hip dislocation (*p* = 0.001, OR = 4.25, 95% CI: 1.6–11.2). They must inform families of the potential risk and closely monitor the patient. Considering that bilateral cases necessitate it, patients must be followed up closely after open reduction, and the clinics’ customary follow-up schemes should be customized to meet the patient’s requirements.

The study presents some limitations. First of all, this study primarily investigated the causes and consequences of bilateral involvement in DDH cases, within the age range of 6–18 months, that necessitated open reduction. Patients aged under six months who were treated with the Pavlik harness were not included in this study. Though we intended to examine the long-term follow-up outcomes of this prospectively followed patient cohort, the age limit specified is an essential limitation. Furthermore, the number of patients in our single-center study may still be inadequate. This restriction implies that our cohort might not have had the necessary power to detect a potential effect, specifically in ascertaining the long-term impacts of bilateral involvement. Further nationwide multifactorial analyses via multicenter studies on the same subject could offer diverse insights on the subject. Secondly, although our average follow-up period can be deemed adequate for evaluation, the requirement of a minimum follow-up period of two years is an important limitation. Moreover, there is a significant limitation in the lack of an objective scoring system (either functional or radiological). On the other hand, we believe we have mitigated this limitation with the fact that all clinical and radiological assessments in our study were performed by the same senior surgeon (AB). Another limitation of our study is that we were unable to evaluate breech presentation as a separate risk factor, instead analyzing it as a subheading of caesarean section. Due to the retrospective nature of our study, we were unable to evaluate this parameter separately. Last but not least, all patients were operated on via the limited posteromedial approach in this study. This is a significant limitation as there are various alternative open reduction techniques available, including the Ferguson, Ludloff, or anterior approaches.

## 5. Conclusions

This study conclusively shows that swaddling infants increases the risk of developing bilateral DDH. The risk of bilateral involvement increases further, particularly in cases of swaddling for more than a month. It is crucial that clinicians, families, and the public are informed about this significant risk factor. Also, orthopedic surgeons treating DDH should be aware that bilateral DDH increases the risk of redislocation significantly following open reduction and should plan the patient’s follow-up process accordingly.

## Figures and Tables

**Table 1:** Risk analysis of main cohort for bilateral involvement.

		Bilateral DDH (N = 173 Patients)	Unilateral DDH (N = 388 Patients)	P
**Sex**	Female	150 (86.7%)	339 (87.4%)	0.828
	Male	23 (13.3%)	49 (12.6%)	
**Number of Births of** **Patient’s Mother**		2 (1) (1–7)	2 (1) (1–10)	0.923
**Birth** **Type**	NVD	135 (78%)	283 (72.9%)	0.201
	C-S	38 (22%)	105 (27.1%)	
**Family History of DDH**	None	94 (54.3%)	230 (59.3%)	0.281
	First D.	34 (19.7%)	56 (14.4%)	
	Other	45 (26%)	102 (26.3%)	
**Consanguineous Marriage**	None	134 (77.5%)	295 (76%)	0.932
	First D.	30 (17.3%)	71 (18.3%)	
	Second D.	9 (5.2%)	22 (5.7%)	
**Use of Swaddling** (Weeks)		4 (16) (0–65)	1 (12) (0–80)	**0.012**
**Use of Swaddling**	<4 weeks	72 (41.6%)	223 (57.5%)	**0.001** **(OR = 1.56,** **95% CI: 1.2–2.0)**
	≥4 weeks	101 (58.4%)	165 (42.5%)	
**Age of Open Reduction** (Months)		10 (11) (6–18)	11 (9) (6–18)	0.713

N: Number of patients; P: Statistical significance value; OR: Odd’s ratio; CI: Confidence Interval; NVD: Normal vaginal birth; C-S: Caesarean section; DDH: Developmental dysplasia of the hip; D.: Degree.

**Table 2:** The long-term outcomes of the developmental hip dysplasia patients in the second cohort.

		Bilateral DDH (N = 276 Hips)	Unilateral DDH (N = 309 Hips)	P
**Sex**	Female	234 (84.8%)	269 (87.1%)	0.429
	Male	42 (15.2%)	40 (12.9%)	
**Age of Open Reduction** (Months)		11.5 (11) (6–18)	11 (9) (6–18)	0.469
**Follow Up** (Years)		9 (8) (2–24)	9 (9) (2–25)	0.993
**Capsule**	Not Opened	75 (27.2%)	90 (29.1%)	0.600
	Opened	201 (72.8%)	219 (70.9%)	
**Last** **Follow-Up Result**	None	185 (67%)	212 (68.6%)	0.832
	RAD	43 (15.6%)	49 (15.9%)	
	AVN	48 (17.4%)	48 (15.5%)	
**Secondary Operation**	None	234 (84.8%)	268 (86.7%)	0.500
	Yes	42 (15.2%)	41 (13.3%)	
**Redislocation**	None	257 (93.1%)	304 (98.4%)	**0.001** **(OR = 4.25,** **95% CI: 1.6–11.2)**
	Yes	19 (6.9%)	5 (1.6%)	
**Osteotomy**	None	253 (91.7%)	273 (88.3%)	0.184
	Yes	23 (8.3%)	36 (11.7%)	

N: Number of patients; P: Statistical significance value; OR: Odd’s ratio; CI: Confidence Interval; RAD: Residual Acetabular Dysplasia; AVN: Avascular necrosis.

## Data Availability

All data have been deposited in a repository.
